# Supplementary material for: Sequential vaccinations with divergent H1N1 influenza virus strains induce multi-H1 clade neutralizing antibodies in swine
Source: Nat Commun. 2023 Nov 27;14:7745. doi: 10.1038/s41467-023-43339-3 (PMC10679120; doi:10.1038/s41467-023-43339-3)
Supplement: Supplementary file 1 — Supplementary Information [file 41467_2023_43339_MOESM1_ESM.pdf]

Supplementary Table 1. Genbank accession numbers for the HA1 and NA sequences of influenza A virus strains used for vaccination (vax), challenge (ch) and serology

	Virus strain	Abbreviation	Subtype	Host	H1 clade		HA accession	N1 lineage	NA accession
					Colloquial	Global nomenclature			
vax	A/California/04/2009	CA09	H1N1	Human	H1N1pdm09	1A.3.3.2	FJ966082	pdm09	FJ969517
	A/swine/Cotes d'Armor/0046/2008	ARM08	H1N1	Swine	EuH1huN1	1B.1.2.3	FN646099	avian	FN646093
	A/swine/Illinois/00685/2005	IL05	H1N1	Swine	US δ2-clade	1B.2.1	FJ638298	human	FJ638300
	A/swine/Gent/28/2010	G10	H1N1	Swine	EuH1avN1	1C.2.1	KP406525	avian	KP406528
ch	A/swine/Ohio/511445/2007	OH07	H1N1	Swine	US γ-clade	1A.3.3.3	EU604689	classical	EU604690
	A/swine/Italy/7704/2001	IT01	H1N1	Swine	EuH1huN1	1B.1.2.2	GQ175960	avian	FJ975100
serology	A/swine/Gent/26/2012	G12	H1N2	Swine	EuH1huN2	1B.1.2.1	KP406526	n.a.	KP406529
	A/swine/Belgium/1/1983	BE83	H1N1	Swine	EuH1avN1	1C.2.1-like	AF091316	avian	AJ412690
	A/swine/Belgium/1/1998	BE98	H1N1	Swine	EuH1avN1	1C.2	FJ805962	avian	FJ805963
	A/swine/Minnesota/02093/2008	MN08	H1N1	Swine	US α-clade	1A.1.1	HM461794	classical	HM461796
	A/swine/Iowa/A01675568/2016	IA16	H1N2	Swine	US α-clade	1A.1.1	KU761546	n.a.	KU761548
	A/swine/Iowa/H04YS2/2004	IA04	H1N1	Swine	US β-clade	1A.2	GQ452235	classical	GQ452237
	A/swine/Oklahoma/A01732565/2016	OK16	H1N1	Swine	US β-clade	1A.2	KX008705	classical	KX008706
	A/swine/Illinois/A01203922/2012	IL12	H1N1	Swine	US γ2-clade	1A.3.2	KC134375	classical	KC134374
	A/swine/Illinois/A01731240/2016	IL16	H1N1	Swine	US γ-clade	1A.3.3.3	KU861208	classical	KU861209
	A/swine/Minnesota/07002083/2007	MN07	H1N1	Swine	US δ2-clade	1B.2.1	FJ611898	human	FJ611900
	A/swine/Georgia/A01732809/2016	GA16	H1N2	Swine	US δ2-clade	1B.2.1	KX008702	n.a.	KX008703
	A/swine/Colorado/A01555119/2014	CO14	H1N1	Swine	US δ1-clade	1B.2.2.1	KP341381	classical	KP341382
	A/swine/Illinois/A01047020/2010	IL10	H1N2	Swine	US δ1-clade	1B.2.2.2	JQ756323	n.a.	JQ756349
	A/Puerto Rico/8/1934	PR34	H1N1	Human	Human	Human	CY009444	human	EF467823
	A/USSR/90/1977	USSR77	H1N1	Human	Human	Human	CY010372	human	CY010374
	A/Taiwan/1/1986	TW86	H1N1	Human	Human	Human	X17224	human	JF816581
	A/New Caledonia/20/1999	NC99	H1N1	Human	Human	Human	DQ508857	human	DQ508859
	A/Brisbane/59/2007	BR07	H1N1	Human	Human	Human	CY058487	human	CY058487
	A/Michigan/45/2015	MI15	H1N1	Human	H1N1pdm09	1A.3.3.2	KY090610	pdm09	KY090612
	A/swine/Missouri/A01410819/2014	MO14	H3N1	Swine	n.a.	n.a.	KJ941380	classical	KJ941381
	A/duck/Minnesota/1525/1981	MN81	H5N1	Duck	n.a.	n.a.	CY014726	avian	/
	A/mallard/Italy/3401/2005	IT05	H5N1	Mallard	n.a.	n.a.	CY021397	avian	CY021399
	A/swine/Gent/172/2008	G08	H3N2	Swine	n.a.	n.a.	KX092355	n.a.	KC142128
	A/swine/Pennsylvania/A01076777/2010	PA10	H3N2	Swine	n.a.	n.a.	JF263535	n.a.	JF312066
	A/mallard/Alberta/205/1998	AB98	H2N3	Mallard	n.a.	n.a.	CY003968	n.a.	CY003970
	A/chicken/Belgium/150/1999	BE99	H5N2	Chicken	n.a.	n.a.	FJ750571	n.a.	FJ750569
	A/chicken/Italy/22A/1998	IT98	H5N9	Chicken	n.a.	n.a.	CY022621	n.a.	CY022623
	A/chicken/Italy/1067/1999	IT99	H7N1	Chicken	n.a.	n.a.	GU052938	avian	AJ416627

n.a.: not applicable

Supplementary Table 2. P sequence values (upper right triangle) and P all antigenic site values (lower left triangle) between the HA1 of H1 influenza A virus (IAV) strains

Virus strain	Vaccine strains				European swine				North American swine											Human					
	CA09 (1A.3.3.2)	ARM08 (1B.1.2.3)	IL05 (1B.2.1)	G10 (1C.2.1)	G12 (1B.1.2.1)	IT01 (1B.1.2.2)	BE83 (1C.1.2)	BE98 (1C.2)	MN08 (1A.1.1)	IA16 (1A.1.1)	IA04 (1A.2)	OK16 (1A.2)	IL12 (1A.3.2)	OH07 (1A.3.3.3)	IL16 (1A.3.3.3)	MN07 (1B.2.1)	GA16 (1B.2.1)	CO14 (1B.2.2.1)	IL10 (1B.2.2.2)	PR34 (human)	USSR77 (human)	TW86 (1B.2)	NC99 (1B.2)	BR07 (1B.2)	MI15 (1A.3.3.2)
CA09 (1A.3.3.2)		0.281	0.273	0.266	0.291	0.272	0.232	0.260	0.144	0.162	0.113	0.150	0.150	0.092	0.116	0.273	0.290	0.270	0.279	0.242	0.269	0.266	0.276	0.276	0.037
ARM08 (1B.1.2.3)	0.540		0.138	0.297	0.101	0.073	0.275	0.291	0.287	0.303	0.284	0.309	0.306	0.291	0.291	0.138	0.176	0.153	0.141	0.172	0.098	0.089	0.129	0.156	0.291
IL05 (1B.2.1)	0.500	0.360		0.282	0.150	0.117	0.252	0.264	0.276	0.273	0.270	0.285	0.285	0.273	0.279	0.025	0.068	0.089	0.077	0.169	0.123	0.077	0.028	0.049	0.285
G10 (1C.2.1)	0.320	0.600	0.520		0.309	0.287	0.122	0.052	0.248	0.266	0.232	0.251	0.281	0.260	0.257	0.276	0.281	0.282	0.279	0.264	0.260	0.266	0.267	0.273	0.281
G12 (1B.1.2.1)	0.560	0.180	0.300	0.620		0.095	0.278	0.294	0.291	0.309	0.287	0.306	0.312	0.284	0.287	0.147	0.182	0.184	0.166	0.199	0.113	0.104	0.141	0.163	0.303
IT01 (1B.1.2.2)	0.560	0.180	0.260	0.580	0.100		0.254	0.272	0.272	0.294	0.272	0.287	0.284	0.263	0.266	0.117	0.151	0.153	0.138	0.172	0.083	0.061	0.107	0.135	0.287
BE83 (1C.1.2)	0.360	0.640	0.560	0.240	0.660	0.620		0.098	0.208	0.232	0.202	0.214	0.232	0.214	0.211	0.252	0.250	0.264	0.255	0.224	0.232	0.235	0.242	0.252	0.232
BE98 (1C.2)	0.280	0.580	0.480	0.100	0.580	0.540	0.180		0.235	0.251	0.229	0.251	0.275	0.254	0.248	0.270	0.265	0.273	0.276	0.248	0.251	0.251	0.258	0.264	0.269
MN08 (1A.1.1)	0.260	0.540	0.500	0.340	0.560	0.540	0.340	0.300		0.144	0.119	0.168	0.171	0.128	0.153	0.279	0.287	0.285	0.291	0.233	0.257	0.266	0.279	0.282	0.147
IA16 (1A.1.1)	0.360	0.620	0.560	0.480	0.600	0.620	0.460	0.420	0.300		0.153	0.187	0.174	0.153	0.153	0.282	0.287	0.294	0.301	0.242	0.291	0.281	0.276	0.279	0.150
IA04 (1A.2)	0.160	0.560	0.500	0.280	0.560	0.540	0.340	0.260	0.240	0.320		0.076	0.144	0.107	0.125	0.273	0.290	0.276	0.273	0.224	0.251	0.260	0.267	0.267	0.128
OK16 (1A.2)	0.200	0.620	0.500	0.280	0.580	0.560	0.300	0.280	0.320	0.360	0.160		0.165	0.141	0.153	0.291	0.299	0.294	0.294	0.239	0.278	0.281	0.282	0.282	0.162
IL12 (1A.3.2)	0.320	0.540	0.480	0.440	0.560	0.540	0.420	0.420	0.380	0.420	0.340	0.340		0.128	0.135	0.285	0.299	0.301	0.291	0.230	0.275	0.278	0.282	0.282	0.141
OH07 (1A.3.3.3)	0.120	0.620	0.500	0.360	0.580	0.560	0.380	0.340	0.220	0.380	0.180	0.220	0.280		0.043	0.267	0.287	0.279	0.282	0.227	0.248	0.257	0.270	0.264	0.089
IL16 (1A.3.3.3)	0.180	0.640	0.500	0.380	0.600	0.580	0.340	0.340	0.260	0.400	0.240	0.240	0.280	0.100		0.273	0.293	0.285	0.291	0.230	0.257	0.260	0.276	0.273	0.116
MN07 (1B.2.1)	0.500	0.360	0.020	0.520	0.320	0.280	0.560	0.480	0.500	0.560	0.500	0.520	0.480	0.500	0.500		0.080	0.089	0.077	0.175	0.120	0.077	0.028	0.049	0.288
GA16 (1B.2.1)	0.500	0.420	0.160	0.520	0.360	0.300	0.560	0.480	0.520	0.580	0.520	0.520	0.500	0.520	0.520	0.180		0.139	0.114	0.201	0.157	0.120	0.080	0.099	0.293
CO14 (1B.2.2.1)	0.460	0.320	0.140	0.520	0.380	0.360	0.580	0.500	0.500	0.580	0.500	0.540	0.520	0.540	0.560	0.140	0.240		0.074	0.202	0.144	0.110	0.083	0.098	0.288
IL10 (1B.2.2.2)	0.500	0.300	0.180	0.540	0.340	0.320	0.600	0.540	0.540	0.620	0.520	0.560	0.500	0.540	0.560	0.180	0.220	0.140		0.187	0.135	0.104	0.058	0.077	0.288
PR34 (human)	0.460	0.460	0.480	0.480	0.500	0.500	0.520	0.460	0.440	0.460	0.500	0.480	0.420	0.500	0.500	0.480	0.520	0.480	0.520		0.138	0.150	0.160	0.181	0.248
USSR77 (human)	0.540	0.180	0.300	0.540	0.140	0.120	0.580	0.480	0.520	0.600	0.500	0.560	0.520	0.540	0.560	0.300	0.360	0.320	0.320	0.460		0.061	0.107	0.132	0.284
TW86 (1B.2)	0.540	0.240	0.180	0.560	0.180	0.140	0.580	0.500	0.520	0.600	0.520	0.540	0.520	0.540	0.560	0.200	0.280	0.280	0.280	0.480	0.160		0.061	0.092	0.281
NC99 (1B.2)	0.520	0.360	0.040	0.540	0.300	0.260	0.600	0.520	0.500	0.560	0.520	0.520	0.500	0.520	0.540	0.060	0.200	0.160	0.160	0.460	0.300	0.180		0.031	0.291
BR07 (1B.2)	0.520	0.420	0.080	0.540	0.360	0.320	0.600	0.520	0.520	0.580	0.520	0.520	0.500	0.520	0.540	0.100	0.220	0.160	0.160	0.500	0.320	0.240	0.060		0.291
MI15 (1A.3.3.2)	0.080	0.560	0.560	0.380	0.620	0.620	0.400	0.340	0.280	0.320	0.240	0.260	0.300	0.160	0.220	0.560	0.560	0.520	0.560	0.460	0.600	0.600	0.580	0.580	

P sequence is defined as: Number of aa substitutions in the HA1 domain of HA / Total number of aa in the HA1 domain of HA (326 or 327 aa). P all antigenic site is defined as: Number of aa substitutions in all five antigenic sites of the HA1 / Total number of aa in all five antigenic sites of the HA1 [56]. A total of 326 or 327 aa residues were examined of which 50 were located in antigenic sites as defined by Caton et al. [3]. The four vaccine strains are shown first, followed by swine IAV strains from Europe and North America and human seasonal IAV strains.

Supplementary Table 3. Hemagglutination inhibition (HI) titers used for the calculation of antigenic distances between H1 strains

	HI antibody titer with monospecific antiserum against influenza A virus strains from																							
	Vaccines				European swine				North American swine											Humans				
Virus strain	CA09 (1A.3.3.2)	ARM08 (1B.1.2.3)	IL05 (1B.2.1)	G10 (1C.2.1)	G12 (1B.1.2.1)	IT01 (1B.1.2.2)	BE83 (1C.1.2)	BE98 (1C.2)	MN08 (1A.1.1)	IA16 (1A.1.1)	IA04 (1A.2)	OK16 (1A.2)	IL12 (1A.3.2)	OH07 (1A.3.3.3)	IL16 (1A.3.3.3)	MN07 (1B.2.1)	GA16 (1B.2.1)	CO14 (1B.2.2.1)	IL10 (1B.2.2.2)	PR34 (human)	USSR77 (human)	TW86 (1B.2)	NC99 (1B.2)	BR07 (1B.2)
CA09 (1A.3.3.2)	**5120**	20	10	20	<10	40	320	80	<10	<10	40	10	<10	320	<10	<10	<10	<10	<10	80	10	<10	<10	<10
ARM08 (1B.1.2.3)	20	**640**	<10	<10	80	640	10	<10	<10	<10	10	<10	<10	<10	<10	<10	<10	<10	40	<10	80	80	<10	<10
IL05 (1B.2.1)	<10	20	**2560**	<10	20	160	<10	<10	<10	<10	<10	<10	<10	<10	<10	640	320	20	20	320	20	<10	1280	40
G10 (1C.2.1)	20	10	<10	**640**	<10	<10	80	160	<10	<10	10	<10	<10	40	<10	<10	<10	<10	<10	10	<10	<10	<10	<10
G12 (1B.1.2.1)	<10	640	10	10	**1280**	5120	<10	<10	<10	<10	10	<10	<10	<10	<10	<10	<10	<10	<10	40	80	160	10	<10
IT01 (1B.1.2.2)	10	640	10	10	1280	**5120**	<10	20	<10	<10	10	<10	<10	<10	<10	10	<10	<10	<10	80	160	160	20	<10
BE83 (1C.1.2)	80	20	<10	10	<10	40	**1280**	80	<10	<10	40	<10	80	<10	<10	<10	<10	<10	<10	10	<10	<10	<10	<10
BE98 (1C.2)	160	<10	<10	160	<10	80	640	**5120**	<10	<10	160	10	10	10	20	<10	<10	<10	<10	40	<10	<10	<10	<10
MN08 (1A.1.1)	160	10	10	10	<10	80	640	80	**320**	<10	80	10	10	640	10	10	<10	<10	<10	80	<10	<10	<10	<10
IA16 (1A.1.1)	<10	<10	<10	<10	<10	<10	<10	<10	<10	**80**	<10	<10	<10	<10	<10	<10	<10	<10	<10	<10	<10	<10	<10	<10
IA04 (1A.2)	80	10	<10	40	<10	40	320	80	20	<10	**640**	320	<10	10	10	<10	<10	<10	<10	160	<10	<10	<10	<10
OK16 (1A.2)	80	10	<10	<10	<10	40	80	10	10	<10	80	**160**	<10	40	<10	<10	<10	<10	<10	80	<10	<10	<10	<10
IL12 (1A.3.2)	20	<10	<10	<10	<10	<10	10	<10	<10	<10	10	<10	**640**	<10	<10	<10	<10	<10	<10	20	<10	<10	<10	<10
OH07 (1A.3.3.3)	320	10	10	20	<10	80	640	80	80	<10	80	10	10	**1280**	40	10	<10	<10	<10	80	<10	<10	<10	<10
IL16 (1A.3.3.3)	640	<10	<10	<10	<10	80	160	10	10	<10	40	80	20	640	**80**	10	<10	<10	<10	<10	<10	<10	<10	<10
MN07 (1B.2.1)	<10	10	2560	10	20	320	20	<10	<10	<10	<10	<10	<10	<10	<10	**1280**	640	80	40	160	20	10	2560	160
GA16 (1B.2.1)	<10	<10	80	<10	<10	40	<10	<10	<10	<10	<10	<10	<10	<10	<10	<10	**320**	<10	10	10	<10	<10	10	<10
CO14 (1B.2.2.1)	<10	10	640	<10	<10	10	<10	<10	<10	<10	<10	<10	<10	<10	<10	160	80	**640**	20	20	<10	<10	160	20
IL10 (1B.2.2.2)	<10	10	40	<10	<10	<10	<10	<10	<10	<10	<10	<10	<10	<10	<10	<10	10	10	**1280**	<10	<10	<10	40	160
PR34 (human)	10	10	10	<10	10	80	<10	<10	<10	<10	10	<10	<10	<10	<10	<10	<10	<10	<10	**5120**	<10	<10	<10	<10
USSR77 (human)	<10	20	<10	<10	<10	160	<10	<10	<10	<10	<10	<10	<10	<10	<10	<10	<10	<10	<10	<10	**320**	<10	<10	<10
TW86 (1B.2)	<10	20	10	<10	<10	1280	<10	<10	<10	<10	<10	<10	<10	<10	<10	10	<10	<10	<10	<10	20	**5120**	20	<10
NC99 (1B.2)	<10	<10	320	<10	<10	10	<10	<10	<10	<10	<10	<10	<10	<10	<10	160	<10	<10	20	80	<10	<10	**320**	40
BR07 (1B.2)	<10	<10	160	<10	<10	<10	<10	<10	<10	<10	<10	<10	<10	<10	<10	40	10	<10	20	<10	<10	<10	40	**640**
MI15 (1A.3.3.2)	2560	20	<10	10	<10	80	80	40	<10	<10	40	<10	10	320	<10	<10	<10	<10	<10	80	<10	<10	<10	<10

Values in bold indicate homologous titers in cross-HI assays. Hyperimmune swine sera were used in HI assays against the four vaccine strains and heterologous H1 swine influenza A virus (IAV) strains from Europe and North America. Post-infection ferret sera were used in HI assays against human seasonal IAV strains.

Supplementary Table 4. Antigenic distances between the HA of H1 influenza A virus strains

Virus strain	Vaccine strains				European swine				North American swine											Human					
	CA09 (1A.3.3.2)	ARM08 (1B.1.2.3)	IL05 (1B.2.1)	G10 (1C.2.1)	G12 (1B.1.2.1)	IT01 (1B.1.2.2)	BE83 (1C.1.2)	BE98 (1C.2)	MN08 (1A.1.1)	IA16 (1A.1.1)	IA04 (1A.2)	OK16 (1A.2)	IL12 (1A.3.2)	OH07 (1A.3.3.3)	IL16 (1A.3.3.3)	MN07 (1B.2.1)	GA16 (1B.2.1)	CO14 (1B.2.2.1)	IL10 (1B.2.2.2)	PR34 (human)	USSR77 (human)	TW86 (1B.2)	NC99 (1B.2)	BR07 (1B.2)	MI15 (1A.3.3.2)
CA09 (1A.3.3.2)		6.80	7.72	4.72	7.42	7.40	4.42	4.32	3.54	5.99	4.18	3.90	5.98	2.91	3.38	7.87	6.76	7.45	7.15	6.35	6.27	7.71	7.30	7.16	1.42
ARM08 (1B.1.2.3)			6.36	6.54	2.54	2.84	5.96	7.05	6.79	5.09	6.40	5.88	5.98	6.81	6.05	6.51	5.53	6.54	5.61	6.09	3.35	4.11	6.64	6.25	6.54
IL05 (1B.2.1)				7.12	6.05	6.06	7.26	7.77	7.36	5.46	7.11	6.64	6.18	7.44	7.13	1.33	3.64	2.85	4.90	5.13	5.40	6.22	2.70	3.90	7.62
G10 (1C.2.1)					7.01	7.07	4.37	3.57	4.71	4.55	4.27	4.96	5.27	4.38	4.94	7.10	5.75	6.41	5.91	6.47	5.54	7.08	6.37	5.91	5.11
G12 (1B.1.2.1)						0.91	6.73	7.35	7.19	5.79	6.75	6.39	6.52	7.24	6.81	6.30	5.86	6.76	6.62	5.47	4.17	4.15	6.68	6.71	7.12
IT01 (1B.1.2.2)							6.82	7.24	7.30	5.99	6.82	6.55	6.70	7.34	6.99	6.37	6.11	6.88	6.84	5.48	4.23	4.35	6.69	6.88	7.13
BE83 (1C.1.2)								3.50	4.27	4.98	3.76	4.28	4.18	4.24	4.26	7.21	5.92	6.77	6.31	6.41	5.40	6.85	6.76	6.35	4.56
BE98 (1C.2)									4.51	5.86	3.33	4.85	5.91	4.20	4.96	7.76	6.62	7.42	7.05	6.53	6.26	7.43	7.27	7.06	4.73
MN08 (1A.1.1)										5.46	3.73	3.56	5.54	1.32	3.34	7.47	6.23	7.08	6.83	5.93	5.92	7.24	6.80	6.72	3.48
IA16 (1A.1.1)											5.03	4.44	3.77	5.48	4.83	5.59	3.82	4.70	4.24	5.16	3.81	5.54	4.64	4.14	5.81
IA04 (1A.2)												2.28	5.18	3.75	4.15	7.23	5.89	6.82	6.45	5.08	5.59	6.97	6.49	6.45	4.41
OK16 (1A.2)													4.75	3.55	3.04	6.84	5.36	6.36	6.00	4.76	5.01	6.57	6.06	6.00	3.84
IL12 (1A.3.2)														5.52	4.76	6.38	4.90	5.59	5.22	5.33	4.88	6.45	5.43	5.16	5.60
OH07 (1A.3.3.3)															2.70	7.55	6.27	7.13	6.86	5.95	5.95	7.27	6.86	6.75	2.84
IL16 (1A.3.3.3)																7.18	5.67	6.62	6.21	6.28	5.26	6.50	6.51	6.11	3.09
MN07 (1B.2.1)																	3.91	2.62	4.72	5.66	5.54	6.13	2.90	3.64	7.83
GA16 (1B.2.1)																		3.72	4.30	5.31	4.33	5.65	4.41	4.10	6.61
CO14 (1B.2.2.1)																			4.14	5.91	5.47	6.34	3.06	3.50	7.41
IL10 (1B.2.2.2)																				6.19	5.14	6.32	3.92	2.83	7.08
PR34 (human)																					5.31	6.55	5.25	6.15	6.00
USSR77 (human)																						4.61	5.49	5.14	6.09
TW86 (1B.2)																							6.31	6.23	7.47
NC99 (1B.2)																								2.72	7.26
BR07 (1B.2)																									7.15
MI15 (1A.3.3.2)																									

Antigenic distances were calculated as described by Peeters et al. 2017 [59] and expressed in antigenic units.

Supplementary Table 5. P sequence values (upper right triangle) and P all antigenic site values (lower left triangle) between the NA of N1 influenza A virus (IAV) strains

Virus strain	Vaccine strains				European swine		North American swine					Human		Avian	
	CA09	ARM08	IL05	G10	BE83	IT01	OH07	IL12	MO14	OK16	MN07	PR34	USSR77	IT05	IT99
CA09		0.090	0.183	0.092	0.072	0.068	0.168	0.179	0.179	0.194	0.188	0.172	0.168	0.102	0.103
ARM08	0.073		0.179	0.034	0.079	0.077	0.179	0.205	0.201	0.200	0.183	0.170	0.175	0.104	0.096
IL05	0.176	0.155		0.186	0.154	0.168	0.188	0.203	0.207	0.200	0.017	0.112	0.083	0.149	0.136
G10	0.067	0.031	0.171		0.090	0.085	0.183	0.211	0.209	0.207	0.186	0.172	0.177	0.113	0.105
BE83	0.067	0.057	0.140	0.073		0.051	0.147	0.171	0.167	0.181	0.158	0.137	0.141	0.058	0.051
IT01	0.057	0.052	0.161	0.057	0.052		0.164	0.183	0.177	0.190	0.173	0.150	0.149	0.079	0.078
OH07	0.155	0.161	0.155	0.166	0.140	0.150		0.087	0.090	0.107	0.192	0.165	0.171	0.156	0.154
IL12	0.181	0.192	0.166	0.202	0.176	0.187	0.088		0.015	0.136	0.207	0.174	0.181	0.181	0.183
MO14	0.181	0.192	0.171	0.202	0.176	0.181	0.093	0.021		0.130	0.207	0.170	0.184	0.179	0.179
OK16	0.166	0.166	0.150	0.176	0.145	0.155	0.073	0.114	0.098		0.196	0.176	0.194	0.183	0.177
MN07	0.187	0.171	0.016	0.181	0.155	0.176	0.166	0.176	0.176	0.150		0.115	0.089	0.154	0.141
PR34	0.166	0.145	0.093	0.161	0.124	0.150	0.150	0.155	0.145	0.140	0.104		0.079	0.134	0.128
USSR77	0.171	0.161	0.073	0.176	0.155	0.166	0.145	0.161	0.161	0.150	0.088	0.083		0.145	0.136
IT05	0.104	0.098	0.130	0.114	0.067	0.098	0.140	0.171	0.176	0.145	0.145	0.124	0.155		0.022
IT99	0.098	0.083	0.114	0.098	0.052	0.083	0.140	0.171	0.171	0.135	0.130	0.119	0.140	0.031	

P sequence is defined as: Number of aa substitutions in the NA / Total number of aa in the NA (469 or 470 aa). P all antigenic site is defined as: Number of aa substitutions in putative antigenic sites of the NA / Total number of aa in putative antigenic sites of the NA [49]. A total of 469 or 470 aa residues were considered of which 193 were located in antigenic sites as defined by Maurer-Stroh et al. 2009 [58]. The four vaccine strains are shown first, followed by swine IAV strains from Europe and North America, human seasonal and avian IAV strains.

Supplementary Table 6. Neuraminidase inhibition (NI) titers used for the calculation of antigenic distances between N1 strains

Virus strain	NI antibody titer with monospecific antiserum against influenza A virus strains from												
	Vaccines				European swine		North American swine					Humans	
	CA09	ARM08	IL05	G10	BE83	IT01	OH07	IL12	MO14	OK16	MN07	PR34	USSR77
CA09	**20480**	80	80	80	5120	160	640	<10	<10	40	40	160	<10
ARM08	640	**1280**	20	160	2560	2560	10	<10	<10	<10	10	<10	80
IL05	40	80	**5120**	<10	160	1280	40	<10	<10	<10	2560	640	40
G10	1280	320	20	**2560**	5120	10	160	<10	<10	<10	10	10	<10
BE83	1280	80	20	80	**10240**	80	80	160	<10	<10	10	40	<10
IT01	320	320	20	20	640	**5120**	20	<10	<10	20	20	80	160
OH07	2560	10	80	40	5120	320	**5120**	20	10	320	80	160	<10
IL12	160	10	40	10	640	80	40	**5120**	160	10	80	160	<10
MO14	640	<10	40	<10	640	<10	40	5120	**5120**	<10	<10	80	<10
OK16	1280	10	80	20	5120	320	640	40	10	**640**	40	160	20
MN07	40	20	10240	10	320	640	80	<10	<10	<10	**5120**	320	10
PR34	<10	20	20	<10	160	320	<10	<10	<10	<10	<10	**20840**	<10
USSR77	<10	80	10	<10	20	640	<10	<10	<10	<10	<10	20	**640**

Values in bold indicate homologous titers in cross-NI assays. Hyperimmune swine sera were used in NI assays against the four vaccine strains and heterologous N1 swine influenza A virus (IAV) strains. Post-infection ferret sera were used in NI assays against human seasonal IAV strains.

Supplementary Table 7. Antigenic distances between the NA of N1 influenza A virus strains

Virus strain	Vaccine strains				European swine		North American swine					Human	
	CA09	ARM08	IL05	G10	BE83	IT01	OH07	IL12	MO14	OK16	MN07	PR34	USSR77
CA09		3.38	4.83	2.65	2.78	3.66	2.20	5.79	5.63	2.70	4.67	5.02	5.08
ARM08			4.36	2.87	2.91	1.70	4.34	5.45	5.65	4.05	4.64	4.62	2.93
IL05				5.35	5.40	3.79	4.92	5.69	6.33	4.84	1.11	3.82	3.76
G10					2.40	3.99	3.75	5.62	5.33	4.03	5.20	5.37	4.81
BE83						3.76	3.53	4.34	4.65	3.45	5.32	5.05	4.80
IT01							4.21	5.59	5.99	3.71	4.29	3.76	2.12
OH07								5.29	5.44	1.48	4.56	5.05	5.19
IL12									2.59	4.96	5.61	5.31	5.36
MO14										5.25	6.29	5.76	5.70
OK16											4.67	4.71	4.60
MN07												4.27	4.43
PR34													3.83
USSR77													

Antigenic distances were calculated as described by Peeters et al. 2017 [59] and expressed in antigenic units.

Supplementary Table 8. Summary of serologic cross-reactivity against antigenically diverse H1 and N1 influenza A virus (IAV) strains

Experiment	Prime-boost	Group	*N* pigs	% of strains for which antibody titers were ≥ threshold in each assay					
				HI (*n* = 24)		VN (*n* = 24)		NI (*n* = 14)	
				≥40	≥640	≥64	≥1024	≥160	≥2560
1	Ctrl.	PBS-PBS	10	0.0	0.0	0.0	0.0	0.0	0.0
	Hom.	CA09-CA09	10	37.5	12.5	33.3	12.5	71.4	14.3
		ARM08-ARM08	5	25.0	12.5	20.8	12.5	21.4	0.0
		IL05-IL05	10	29.2	12.5	29.2	12.5	14.3	14.3
		G10-G10	5	16.7	4.2	12.5	4.2	14.3	7.1
	Het.	CA09-G10	5	41.7	4.2	41.7	8.3	50.0	7.1
		G10-CA09	5	29.2	4.2	29.2	0.0	64.3	21.4
		CA09-IL05	5	29.2	0.0	33.3	0.0	78.6	14.3
		ARM08-IL05	5	20.8	8.3	20.8	8.3	21.4	0.0
		IL05-ARM08	5	16.7	0.0	20.8	0.0	28.6	0.0
		CA09-ARM08	5	50.0	4.2	50.0	0.0	42.9	0.0
		G10-IL05	5	0.0	0.0	0.0	0.0	35.7	0.0
		G10-ARM08	5	0.0	0.0	0.0	0.0	14.3	0.0
2a	Ctrl.	PBS-PBS	2	0.0	0.0	0.0	0.0	0.0	0.0
	Hom.	TIV-TIV	7	50.0	4.2	41.7	0.0	42.9	0.0
	Het.	G10-ARM08+CA09	7	29.2	4.2	29.2	0.0	50.0	7.1
2b	Ctrl.	3xPBS	4	0.0	0.0	0.0	0.0	0.0	0.0
	Hom.	3xARM08	7	20.8	12.5	20.8	12.5	35.7	0.0
		3xTIV	7	62.5	37.5	62.5	37.5	71.4	28.6
	Het.	G10-ARM08-CA09	7	75.0	54.2	70.8	45.8	92.9	57.1

Pigs were given two vaccinations with 4 weeks interval (experiment 1 and 2a), and a third vaccination 6 weeks after the second (experiment 2b). Ctrl.: mock-vaccinated control groups; hom.: homologous prime-boost groups; het.: heterologous prime-boost groups. HI, VN and NI antibody titers were determined on pooled sera of each group, 14 days after the final vaccination. Twenty-four antigenically distinct H1 virus strains were used in HI and VN assays, 14 N1 virus strains were used in NI assays. Antibody titers against each strain are given in a Source Data file and represented as heatmaps in Fig. 3.

Supplementary Table 9. Lung and tracheal pathology after challenge with A/swine/Ohio/511445/07 (OH07) or A/swine/Italy/7704/01 (IT01)

			Mean lesion score (number of pigs with lesions)							
			OH07 challenge				IT01 challenge			
					Microscopic lesions				Microscopic lesions	
Experiment	Prime-boost	Group	*n*	Macroscopic pneumonia (%)	Lung (max. 10)	Trachea (max. 2)	*n*	Macroscopic pneumonia (100%)	Lung (max. 10)	Trachea (max. 2)
1	Ctrl.	PBS-PBS	5	2.10% (4)	3.60 (5)	0.80 (4)	5	2.50% (4)	1.50 (4)	1.00 (4)
	Hom.	CA09-CA09	5	0.00%*(0)	3.00 (5)	0.20 (1)	5	0.85% (3)	3.90 (5)	1.20 (5)
		ARM08-ARM08	-	-	-	-	5	0.30% (1)	1.60 (5)	0.40 (2)
		IL05-IL05	5	2.95% (5)	5.20 (5)	1.40 (5)	5	3.95% (5)	4.80 (5)	1.00 (5)
		G10-G10	5	0.60% (4)	2.20 (5)	0.60 (3)	-	-	-	-
	Het.	CA09-G10	5	0.80% (4)	3.90 (5)	0.20 (1)	-	-	-	-
		G10-CA09	5	0.00%* (0)	1.20 (4)	0.00 (0)	-	-	-	-
		CA09-IL05	5	0.30% (1)	2.00 (5)	0.20 (1)	-	-	-	-
		ARM08-IL05	-	-	-	-	5	0.40% (2)	1.90 (5)	0.20 (1)
		IL05-ARM08	-	-	-	-	5	0.70% (3)	1.70 (5)	0.20 (1)
		CA09-ARM08	-	-	-	-	5	0.00% (0)	0.90 (4)	0.60 (3)
2a	Ctrl.	PBS-PBS	2#	1.50% (1)	3.25 (2)	0.50 (1)	2	5.00% (1)	0.50 (1)	0.00 (0)
	Hom.	TIV-TIV	4	0.00% (0)	1.25 (3)	0.25 (1)	3	0.00% (0)	0.83 (2)	1.00 (3)
		G10-ARM08+CA09	4	0.50% (1)	0.50 (2)	0.25 (1)	3	0.00% (0)	2.00 (3)	0.67 (1)
2b	Ctrl.	3xPBS	2	0.20% (2)	0.50 (1)	0.00 (0)	2	3.38% (2)	3.25 (2)	0.00 (0)
	Hom.	3xARM08	4	1.80% (3)	1.62 (3)	0.25 (1)	3	0.00% (0)	0.00 (0)	1.00 (2)
		3xTIV	4	0.20% (2)	0.88 (3)	0.00 (0)	3	0.73% (2)	0.67 (2)	0.00 (0)
	Het.	G10-ARM08-CA09	4	1.40% (2)	0.88 (2)	0.00 (0)	3	0.33% (1)	1.17 (2)	0.33 (1)

Ctrl.: mock-vaccinated control group; hom.: homologous prime-boost groups; het.: heterologous prime-boost groups.
Macroscopic pneumonia was estimated visually for each lung lobe and the percentage of the total lung surface affected with pneumonia was calculated, based on weighted proportions of each lobe to the total volume [67].
Individual microscopic lung lesion scores were assigned based on the severity of three parameters [68]: (1) epithelial damage in intrapulmonary airways (0-3), (2) peribronchiolar lymphocytic cuffing (0-3), (3) neutrophil exudation in bronchioles and alveoli (0-2). For each pig, a composite score was calculated using the sum of the three individual scores. The average group composite score is shown in the table and was used for statistical analysis. Microscopic tracheal lesion scores are based on the severity of epithelial damage (0-2).
Unvaccinated, unchallenged control pigs (*n* = 4) had a mean macroscopic pneumonia score of 0 and mean microscopic lesion scores of 2 (lung) and 0 (trachea).
Asterisks indicate significant differences with the unvaccinated challenge control group (p = 0.037, Kruskal-Wallis test).
#Because of a shortage of pigs in experiment 2a, the table shows scores of two randomly selected unvaccinated challenge control pigs from experiment 1.
