# Supplementary material for: Sequential vaccinations with divergent H1N1 influenza virus strains induce multi-H1 clade neutralizing antibodies in swine
Source: Nat Commun. 2023 Nov 27;14:7745. doi: 10.1038/s41467-023-43339-3 (PMC10679120; doi:10.1038/s41467-023-43339-3)
Supplement: Supplementary file 3 — Description of Additional Supplementary Information [file 41467_2023_43339_MOESM3_ESM.pdf]

Description of Additional Supplementary Items

File name: Supplementary Data 1
Description: P values are shown for HI and VN titers that are statistically higher (twosided $p < 0.05$, Generalized Linear Model with y-intercept suppressed and threshold subtracted from log transformed titers) than the seroprotective threshold (HI titers ≥40, VN titers ≥64), and NI titers that are statistically higher than 160. Actual antibody titers are shown in Table 1.
