# Supplementary material for: Sequential vaccinations with divergent H1N1 influenza virus strains induce multi-H1 clade neutralizing antibodies in swine
Source: Nat Commun. 2023 Nov 27;14:7745. doi: 10.1038/s41467-023-43339-3 (PMC10679120; doi:10.1038/s41467-023-43339-3)
Supplement: Supplementary file 4 — Supplementary Data [file 41467_2023_43339_MOESM4_ESM.pdf]

Supplementary Data 1. *P* values for pre-challenge antibody titers against vaccine and challenge (ch) strains in hemagglutination inhibition (HI), virus neutralization (VN) and neuraminidase inhibition (NI) assays.

			HI						VN						NI					
			Vaccine strains				Ch strains		Vaccine strains				Ch strains		Vaccine strains				Ch strains	
Experiment	Prime-boost	Group	CA09 (1A.3.3.2)	ARM08 (1B.1.2.3)	IL05 (1B.2.1)	G10 (1C.2.1)	OH07 (1A.3.3.3)	IT01 (1B.1.2.2)	CA09 (1A.3.3.2)	ARM08 (1B.1.2.3)	IL05 (1B.2.1)	G10 (1C.2.1)	OH07 (1A.3.3.3)	IT01 (1B.1.2.2)	CA09 (pdm)	ARM08 (av)	IL05 (hu)	G10 (av)	OH07 (1A.3.3.3)	IT01 (1B.1.2.2)
1	Ctrl.	PBS-PBS																		
	Hom.	CA09-CA09	< 2e-16						< 2e-16				6,69E-10		< 2e-16	0,000123		< 2e-16	6,91E-15	
		ARM08-ARM08		1,34E-09						< 2e-16				9,13E-12		1,78E-08				
		IL05-IL05			< 2e-16						< 2e-16						< 2e-16			
		G10-G10				0,014270						0,000117						8,09E-06		
	Het.	CA09-G10	0,000434						< 2e-16				0,000456		1,52E-10			1,52E-10		
		G10-CA09				0,014270									1,29E-13	< 2e-16		1,29E-13		
		CA09-IL05							< 2e-16						1,41E-12	1,78E-08		1,41E-12		
		ARM08-IL05		3,69E-11	0,044886					< 2e-16										
		IL05-ARM08									0,028150									
		CA09-ARM08							3,70E-11						0,000043			0,000043		
		G10-IL05														0,020554		0,040262		
		G10-ARM08																		
2a	Ctrl.	PBS-PBS																		
	Hom.	TIV-TIV	< 2e-16	0,002290		0,000054			1,41E-13	4,70E-10		0,011576			5,08E-06	0,000529		5,08E-06	0,005630	
	Het.	G10-ARM08+CA09	< 2e-16			6,64E-09			2,67E-09			0,000138	0,001340		0,005939	1,85E-11		0,005939	0,000022	
2b	Ctrl.	3xPBS																		
	Hom.	3xARM08		< 2e-16						< 2e-16				2,27E-16		0,006404				0,006316
		3xTIV	< 2e-16	< 2e-16		< 2e-16			< 2e-16	< 2e-16		3,34E-12	2,19E-11	2,19E-13	< 2e-16	0,000029		0,000005	2,32E-14	0,000010
	Het.	G10-ARM08-CA09	< 2e-16	< 2e-16		< 2e-16			< 2e-16	< 2e-16		< 2e-16	3,93E-14	< 2e-16	< 2e-16	< 2e-16		< 2e-16	< 2e-16	2,77E-16

P values are shown for HI and VN titers that are statistically higher (two-sided $p < 0.05$, Generalized Linear Model with y-intercept suppressed and threshold subtracted from log-transformed titers) than the seroprotective threshold (HI titers ≥40, VN titers ≥64), and NI titers that are statistically higher than 160. Actual antibody titers are shown in Table 1.
